# The 5-Es: a model for providing care for non-neurotypical patients

**DOI:** 10.1038/s41390-020-0810-0

**Published:** 2020-02-13

**Authors:** Michele Kong, James Willig

**Affiliations:** The University of Alabama at Birmingham, Birmingham, AL USA

In business, a “closed decision-making system” refers to an environment where all variables, alternatives, and anticipated outcomes are known. In medicine, we traffic in “open decision-making systems”, where we are challenged by successive unknown environments (patients) and lack insight into all available alternatives and anticipated outcomes. We approach these complex open systems with data collection tools granted to us by our training, at the core of which lies the history and physical exam (H&P). History taking is our oldest diagnostic tool and can provide critical insights into the chief presenting complaint. The physical exam adds further diagnostic power, particularly when focused on testing diagnostic hypotheses generated during history taking.^1,2^ Beyond the H&P, our arsenal to navigate complex open decision-making system includes measurement and continual monitoring of biometrics, such as vital signs, laboratory, and imaging studies. A skilled clinician will reorder these data and produce a differential diagnosis by combining and recombining the collected variables into different configurations, then carefully and probabilistically gauge their fit over the presenting pattern to offer an initial diagnosis and treatment plan. Clinicians will train for years to achieve competency in this process, and a lifetime of practice to approach mastery.

This orderly process of data collection is challenged when clinicians encounter non-neurotypical patients, for instance, those with autism spectrum disorder (ASD), or others with developmental delay or learning disabilities when they present with acute illness. This vulnerable, often poorly understood patient population, has chronic health needs and additional barriers (developmental, social, and communication).^3,4^ These characteristics further obfuscate an already challenging open decision-making environment with neurotypical patients, adding additional layers of complexity to the diagnostic process. We share our “5-E” approach to help clinicians navigate such complex situations (*Establish* baseline, *Environmental* adaptation, *Engage* appropriately, *Effectively* communicate, and *Employ* other tools cost-effectively) to reach a diagnosis through a case example:

“A 10-year-old non-verbal female with ASD delay presented to an Emergency Department (ED) with a chief complaint of vomiting, fever and increased agitation. The parents had limited history to contribute. She became increasingly agitated and began striking her head. She was combative making the physical exam difficult. The exam seemed non-focal, and laboratory studies revealed leukocytosis. After provision of intravenous fluids, the patient was discharged home with a presumed diagnosis of viral gastroenteritis.”

*Establish* baseline: In the care of non-neurotypical patients with developmental delay and acute illness, history can be limited and non-specific. The patient’s intrinsic characteristics (limited communication, social impairment, etc.) can also impede the physical exam and biometric monitoring. For instance, a neurotypical patient with an intracranial pathology may be able to express a headache and verbalize associated symptoms. In a developmentally delayed, or non-speaking patient, such history will be absent, and presenting complaints may be vague with non-specific symptoms. It is important to focus on the behavior and to *establish* the patient’s baseline versus presenting behaviors. Questions directed at the patient and caregivers should include:

1. What is the patient’s baseline behavior?
2. Is the presenting behavior new or a change from baseline?

*Environmental* adaptation: Performing a physical exam can be challenging in a patient with abnormal sensory behavior. The hospital environment’s constant and unfamiliar sensory influx (bright lights, noises, smells, etc.), change in routine, new location, and exposure to new people may lead to sensory overload that manifests as a behavioral outburst or agitation.^5^ Environmental adaptation is crucially important to diminish sensory overload; for example, place the patient in a quiet room or use noise-cancelling headphones, turn down the lights, and limit the number of providers.^6,7^

*Engage* appropriately: Engage the patient based on their preferences and functional age. Is the 10-year-old patient at a functional age of 5 years? Does the patient prefer to be held by the parent during an examination? Is being allowed to touch an instrument before an exam calming? The provider needs to acquire and leverage this knowledge to engage the patient appropriately, to facilitate a comprehensive exam.

*Effective* communication: It is important to establish whether the patient is a verbal communicator or uses an augmentative communication device. Communication strategies should always include use of simple, short, and direct instructions. Avoid giving confusing multi-step instructions. It is also important to give the patient time to respond. In many of these patients, identifying their preferred item, using those as motivators, and strategically using encouragement are useful communication techniques. Displaying relaxed body language while paying attention to the patient’s body language can provide important clinical clues. Providers should also always elicit input from parents early on and rely on their insights for best communication strategies with their child.

*Employ* other tools cost-effectively: Despite these adjustments, the data collected through the H&P may remain limited. The placement of devices enabling continual monitoring of biometrics may not be accepted by patients, further limiting available data. Clinicians must thoughtfully and cost-effectively *employ* other diagnostic tools (i.e., laboratory, imaging, etc.) to identify important pathology.

Our patient returned the following day with shock and further investigation revealed bacterial meningoencephalitis. The “head hitting” and increased agitation were not part of the patient’s baseline, yet they were dismissed as part of her ASD by the initial providers. Establishment of baseline with the family by asking targeted behavior questions, adapting the environment, engagement of the patient at her functional age, and use of her communication board are all strategies that may have contributed to the early recognition of the “head hitting” as a new symptom. The resulting, more complete illness script may have resulted in a higher suspicion for intracranial infection and a more aggressive initial diagnostic and therapeutic strategy. Most importantly, it may have provided the opportunity to prevent central nervous system damage, the regrettable neurologic sequelae of this case.

It is critical for medical providers in any setting, primary or acute care, across pediatric and adult care, to understand the unique challenges involved in using our traditional diagnostic toolset in a non-neurotypical population. These sensory, communication, and social barriers to medical care are present from childhood and can become even more magnified into adulthood. If the unique needs of these patients are underappreciated or undervalued, we place patient care and safety at risk. If any of the tools in our diagnostic armamentarium (H&Ps, biometric monitoring, laboratory/imaging studies, etc.) are compromised, skilled clinicians can adapt. In these patient encounters, we offer colleagues the 5-Es. Remember to: (1) *Establish* baseline, (2) *Environmental* adaptation, (3) *Engage* appropriately, (4) *Effectively* communicate, and (5) *Employ* other tools cost-effectively, to reach a diagnosis. Using the 5-Es will help clinicians elicit information required to approach the diagnosis and management of acute illness in the complex, open decision-making system of providing optimal care to non-neurotypical patients.
